# Organization and Quality of Emergency Thoracic Surgical Care in Multidisciplinary Hospitals of Almaty, Kazakhstan: A Multicomponent Study Combining Hospital Data, Patient-Reported Experience, and Healthcare Professional Perspectives

**DOI:** 10.3390/healthcare14172828

**Published:** 2026-09-03

**Authors:** Sultan Zhanbyrbaev, Maksut Kulzhanov, Aueskhan Dzhumabekov, Alfiya Igissenova, Gulstan Esetova, Nadira Aitambayeva, Gulbanu Berdiyarova, Nazerke Narymbayeva, Aigul Tazhiyeva

**Affiliations:** 1 Department of Public Health and Social Sciences, Kazakhstan Medical University “KSPH”, Almaty 050060, Kazakhstan; aitambaeva.nadira@gmail.com; 2 Department of Policy and Management and Healthcare, Asfendiyarov Kazakh National Medical University, Almaty 050012, Kazakhstan; kulzhanov.m@kaznmu.kz; 3 Department of Administration, Kazakhstan Medical University “KSPH”, Almaty 050060, Kazakhstan; dr.jumabekov@gmail.com; 4 Department of Microbiology and Virology, Asfendiyarov Kazakh National Medical University, Almaty 050000, Kazakhstan; igisenova.a@kaznmu.kz; 5 Department of Pulmonology, Asfendiyarov Kazakh National Medical University, Almaty 050000, Kazakhstan; 6 Department of Anesthesiology and Resuscitation with a Course in Emergency Medicine, Kazakhstan Medical University “KSPH”, Almaty 050060, Kazakhstan; berdiyarovagulbanu@gmail.com; 7 Department of Healthcare Management, Kazakhstan Medical University “KSPH”, Almaty 050060, Kazakhstan; n.narymbay@gmail.com; 8 Department of Science, Asfendiyarov Kazakh National Medical University, Almaty 050000, Kazakhstan; a.tazhieva@kaznmu.kz

**Keywords:** emergency thoracic surgery, thoracic trauma, healthcare organization, patient-reported experience, multidisciplinary hospitals, multicomponent study

## Abstract

**Highlights:**

**What are the main findings?**

- A multicomponent study integrating retrospective hospital data, patient-reported experiences, and healthcare professionals’ perspectives provided the first comprehensive evaluation of the organization of emergency thoracic surgical care in Almaty, Kazakhstan.
- Hospital data demonstrated significant changes in the pattern of thoracic pathology, accompanied by reduced hospital mortality and shorter length of stay. At the same time, survey findings identified strengths and remaining organizational challenges from both patient and provider perspectives.

**What are the implications of the main findings?**

- Integrating routine hospital data with patient-reported experiences and healthcare professional perspectives provides a more comprehensive framework for evaluating emergency thoracic surgical services.
- The findings support further strengthening of specialized thoracic trauma services, multidisciplinary coordination, staff training, and resource allocation to improve the quality and efficiency of emergency thoracic care.

**Abstract:**

**Background:** Emergency thoracic conditions remain an important cause of hospitalization, emergency surgery, and mortality, requiring well-organized multidisciplinary thoracic surgical services. **Objectives:** To comprehensively evaluate the organization of emergency thoracic surgical care in multidisciplinary hospitals in Almaty using retrospective hospital data together with patient and healthcare professional-reported assessments. **Methods:** A multicomponent study was conducted comprising three complementary components: (1) a retrospective analysis of official hospital data from five multidisciplinary hospitals providing emergency thoracic surgical care in Almaty during 2019–2023; (2) a questionnaire survey of patients evaluating their experiences with emergency thoracic care; and (3) a questionnaire survey of healthcare professionals assessing the organization of emergency thoracic surgical services. Descriptive statistics, Pearson’s χ^2^ test, Fisher’s exact test where appropriate, Spearman’s rank correlation coefficient, and multivariable ordinal logistic regression were used. Statistical significance was defined as *p* < 0.05. **Results:** During 2019–2023, the annual number of hospitalized patients averaged 519.0 ± 31.7. Chest injuries without internal organ involvement accounted for 50.9% of the 2152 cases included in the pathology-specific analysis, and the distribution of the main thoracic pathology groups changed significantly over time (χ^2^ = 158.8, df = 12, *p*< 0.001). Hospital mortality decreased from 3.13% to 2.59%, while the average length of hospital stay declined from 8.74 to 7.72 days. Patient-reported assessments were generally favorable. In the adjusted analysis, treatment type and treatment-related complications were independently associated with overall satisfaction, whereas age, gender and time to diagnosis were not. Healthcare professionals identified equipment availability, staff training, and interdepartmental coordination as areas requiring further improvement. **Conclusions:** Integrating hospital performance indicators with patient-reported experiences and healthcare professional perspectives provided a comprehensive assessment of emergency thoracic surgical care in Almaty. The observed reductions in mortality and length of hospital stay, together with findings from patient and healthcare professional surveys, identify several organizational priorities to strengthen emergency thoracic surgical services further.

## 1. Introduction

Thoracic trauma remains a major contributor to emergency surgical admissions and trauma-related mortality worldwide [1]. The forecast indicates that the number of patients with severe chest injuries is likely to increase by 2045, potentially placing greater pressure on emergency surgical services and specialized thoracic care units [2]. A comprehensive review of current approaches to chest trauma management in emergency medicine demonstrated that modern diagnostic strategies, advanced imaging techniques, appropriate indications for surgical intervention, and the use of emergency department thoracotomy for blunt and penetrating chest injuries are essential components of patient care [3]. In low- and middle-income countries, the effectiveness of trauma care is largely determined by the organization of trauma services, the availability of specialized surgical care, the level of staff training, and patient routing. Infrastructure deficiencies and resource limitations remain important factors influencing traumatic injury outcomes [4]. The major challenges are the unequal distribution of healthcare resources, limited availability of specialized surgical care, and difficulties coordinating patient transfers between healthcare facilities. In these circumstances, improving the system of specialized emergency care is a key area for enhancing the effectiveness of treatment for traumatic injury [5]. Emergency thoracotomy remains a critical intervention for carefully selected patients with severe thoracic trauma, particularly those sustaining penetrating injuries, and can substantially improve survival when performed under appropriate indicators [6,7,8]. Current WSES-AAST recommendations advocate a multidisciplinary approach to thoracic trauma management, with treatment strategies tailored to the mechanism of injury. Compared to blunt trauma, penetrating chest injuries more frequently require surgical intervention and are associated with poorer clinical outcomes [9]. Despite advances in trauma management, variations in the organization of emergency thoracic surgical services continue to influence access to timely diagnosis, surgical treatment, and patient outcomes. Although several studies have described the epidemiology and clinical outcomes of thoracic trauma, considerably less attention has been paid to the organization of emergency thoracic surgical care at the health-system level. In particular, evidence integrating routine hospital data with patient-reported experiences and healthcare professional perspectives remains limited, especially in middle-income countries. Such an integrated evaluation may provide a more comprehensive understanding of both clinical performance and organizational challenges in emergency thoracic surgical services. In Kazakhstan, emergency thoracic surgical care is concentrated in multidisciplinary hospitals serving large urban populations. However, published evidence evaluating the organization, outcomes, and quality of these services remains scarce. Therefore, a comprehensive evaluation of emergency thoracic surgical care requires not only analysis of hospital activity indicators but also assessment of patient experiences and healthcare professionals’ perspectives regarding service organization and quality. Therefore, this study aimed to comprehensively evaluate the organization of emergency thoracic surgical care in multidisciplinary hospitals in Almaty, Kazakhstan, by integrating retrospective hospital data with patient-reported experiences and healthcare professional perspectives using a multicomponent approach.

## 2. Materials and Methods

### 2.1. Study Design

This multicomponent quantitative study combined a retrospective analysis of hospital data with two cross-sectional questionnaire surveys conducted among patients and healthcare professionals. The three components were used to evaluate emergency thoracic surgical care from complementary perspectives: hospital data characterized service activity and clinical outcomes, the patient survey assessed patients’ experiences with the care received, and the healthcare professional survey examined organizational aspects of emergency thoracic surgical care. The three study components were analyzed separately and integrated at the interpretation stage. Retrospective hospital data were used to assess temporal patterns in service activity, case structure, hospital resource utilization, length of stay, and mortality. Patient survey findings provided information on perceived timeliness and quality of diagnosis and treatment, communication, and support during care. Healthcare professional responses were used to assess resource availability, staff qualifications, interdepartmental coordination, and perceived organizational barriers. The findings from the three components were subsequently considered together to identify areas of convergence and complementary evidence. This approach enabled the interpretation of hospital performance indicators alongside patient and healthcare professional assessments of the organization and quality of emergency thoracic surgical care.

#### 2.1.1. Retrospective Hospital Data

A retrospective analysis was performed using official national healthcare reporting data covering the period 2019–2023. The study included five multidisciplinary hospitals in Almaty that provide emergency thoracic surgical care: Central City Clinical Hospital, City Clinical Hospital No. 7, City Clinical Hospital No. 4, City Hospital of Emergency Care, and Almaty Multidisciplinary Clinical Hospital. Population data were obtained from the statistical digest Health of the Population of the Republic of Kazakhstan and Activities of Healthcare Organizations (Committee on Statistics, Astana, Kazakhstan).

#### 2.1.2. Patient Survey Methodology

A cross-sectional questionnaire survey was conducted between 17 May 2023 and 17 May 2024 among patients who received emergency thoracic surgical care in the five participating hospitals. Eligible patients who agreed to participate were recruited voluntarily during hospitalization. The questionnaire comprised items addressing multiple aspects of emergency thoracic care, including time to diagnosis, quality of healthcare delivery, communication with medical personnel, perceived treatment effectiveness, availability of patient support, and overall satisfaction with the care received. Participation was voluntary and anonymous. The patient survey included 540 patients treated for emergency thoracic conditions across the five participating hospitals. The minimum sample size was estimated for a finite population using a 95% CI level, a 5% margin of error, and an expected proportion of 50%. The calculation yielded a minimum sample of 225 patients. The final survey included 262 eligible patients who agreed to participate.

#### 2.1.3. Healthcare Professional Survey

A separate cross-sectional questionnaire survey was conducted between 17 May 2023 and 17 May 2024 among healthcare professionals involved in emergency thoracic surgical care in the five participating hospitals. Physicians and nurses who met the eligibility criteria were invited to participate voluntarily. A total of 123 eligible healthcare professionals agreed to complete the questionnaire and were included in the analysis.

#### 2.1.4. Study Participants

The study consisted of three complementary components. The retrospective component included official hospital data from five multidisciplinary hospitals providing emergency thoracic surgical care in Almaty between 2019 and 2023. The cross-sectional survey included 262 patients who received emergency thoracic surgical care and 123 healthcare professionals involved in the provision of such services.

#### 2.1.5. Patient Survey: Inclusion and Exclusion Criteria

##### Inclusion Criteria

Patients were eligible for participation if they were aged 18 years or older; Received emergency thoracic surgical care in one of the participating multidisciplinary hospitals; were able to understand the questionnaire and provide informed consent; and agreed to participate voluntarily.

##### Exclusion Criteria

Patients were excluded if they were younger than 18 years, were unable to complete the questionnaire because of severe clinical condition or cognitive impairment; declined participation, or returned a questionnaire with insufficient information for inclusion in the survey dataset. Questionnaires with occasional missing responses to individual items were retained for analysis.

#### 2.1.6. Healthcare Professional Survey: Inclusion and Exclusion Criteria

##### Inclusion Criteria

Healthcare professionals were eligible if they were

Physicians and nurses involved in emergency thoracic surgical care; had direct clinical experience in emergency thoracic surgery or related emergency departments; had worked in the participating hospital during the study period; agreed to participate voluntarily.

##### Exclusion Criteria

Healthcare professionals were excluded if they had no direct involvement in emergency thoracic surgical care, were temporary trainees or students, declined participation, or returned a questionnaire with insufficient information for inclusion in the survey dataset. Questionnaires with occasional missing responses to individual items were retained for analysis.

#### 2.1.7. Questionnaire Administration

Two structured questionnaires were developed by the study author specifically for this study: one for patients and one for healthcare professionals. Before the main survey, the questionnaires were pilot-tested in a small group of patients and healthcare professionals from the participating hospitals. Experts also reviewed the questionnaire items to assess their relevance, clarity, and appropriateness to the study objectives. Feedback obtained during pilot testing and expert review was used to refine the questionnaires before the main survey. Internal consistency was assessed using Cronbach’s alpha for conceptually related questionnaire items. The Cronbach’s alpha coefficient was 0.880 for the patient questionnaire and 0.867 for the healthcare professional questionnaire, indicating good internal consistency. Demographic, clinical, multiple-response, and open-ended items were excluded from the reliability analysis because they were not components of the corresponding evaluative scales. Patients completed a structured questionnaire designed to capture their perceptions of emergency thoracic surgical care. The questionnaire was designed to capture patients’ experiences across several components of emergency thoracic care, including the interval to diagnosis, quality of communication with clinical staff, perceptions of diagnostic and treatment procedures, effectiveness of medical management, adequacy of pain relief, access to supportive resources, and the overall evaluation of healthcare services. The healthcare professional questionnaire evaluated organizational aspects of emergency thoracic surgical care, including resource availability, staff qualifications, interdepartmental coordination, barriers to care delivery, and priorities for service improvement. Both questionnaires consisted primarily of closed-ended questions with categorical or ordinal response options and were available in Russian and Kazakh. Participants completed the questionnaire in their preferred language. Patients completed the questionnaire during hospitalization after receiving information about the study and providing written informed consent. Participation was voluntary and anonymous and did not affect the care provided.

### 2.2. Statistical Analysis

Study data were summarized using descriptive statistics. Continuous variables are presented as means with standard deviations (SD), while categorical variables are presented as absolute frequencies and percentages. Associations between categorical variables were evaluated using Pearson’s χ^2^ test. Fisher’s exact test was used when the assumptions of the χ^2^ test were not met due to low expected cell counts. For statistically significant χ^2^ tests, effect sizes were estimated using Cramer’s V and interpreted as negligible (<0.10), small (0.10–0.30), moderate (0.30–0.50), or large (>0.50). Spearman’s rank correlation coefficient (r_s_) was used to evaluate temporal trends and associations involving ordinal variables. In addition to descriptive and bivariate analyses, multivariable ordinal logistic regression was performed for the main evaluative outcomes of the questionnaire surveys to account for potential confounding. For the patient survey, overall satisfaction with the organization of treatment was used as the dependent variable. Age category, gender, time to diagnosis, treatment type, and treatment-related complications were included as covariates. Age category and time to diagnosis were entered as ordinal variables, while treatment type was modeled as a categorical variable with surgical treatment as the reference category. Treatment-related complications were categorized as present (minor or serious) versus absent. For the healthcare professional survey, the overall assessment of the organization of emergency care was used as the dependent variable. Gender, age category, years of professional experience, qualification category, and thoracic surgery specialization were included as covariates. Adjusted odds ratios (aORs) with 95% confidence intervals (CIs) were calculated. Missing responses to individual questionnaire items were handled using available–case analysis. A missing response to a single item did not result in exclusion of the entire questionnaire. The response was excluded only from the analysis of that specific item. Therefore, denominators could vary across questionnaire questions. Questionnaires containing insufficient information for inclusion in the survey dataset were excluded entirely. No imputation of missing values was performed. Multivariable regression analyses were conducted using complete-case analysis. Of the 262 patients, 255 (97.3%) had complete data for all variables included in the patient model. Standardized measures of injury severity and comorbidity burden were not available and therefore could not be included in the adjusted analysis. A sensitivity analysis was performed to assess the robustness of the patient multivariable findings to an alternative outcome specification. Overall satisfaction with the organization of treatment was dichotomized as fully satisfied versus partially/not satisfied and analyzed using multivariable binary logistic regression. The same covariates and complete-case sample (*n* = 255) as in the primary ordinal logistic regression model were used. Statistical analyses were performed using SPSS version 25.0 (IBM Corp., Armonk, NY, USA). A two-sided *p*-value <0.05 was considered statistically significant. Because multiple comparisons were performed across questionnaire items, the corresponding *p*-values were interpreted as exploratory rather than confirmatory.

### 2.3. Ethical Considerations

The study was approved by the Local Ethical Committee of the Kazakhstan Medical University “KSPH” on 17 May 2023 (protocol number #IRB-73-2023).

All participants received written information about the study objectives, study procedures, confidentiality, and protection of personal information before enrolment. Written informed consent was obtained from all participants before participation. Participation was voluntary, and all questionnaire responses were collected anonymously.

## 3. Results

The majority of surgically treated patients were aged 18–59 years (72.9%). Although the proportion of older patients increased over time, the differences between years were not statistically significant (χ^2^ = 20.91, *p* = 0.052). Detailed data are presented in Appendix A, Table A1.

For the period 2019–2023, the average number of hospitalized patients was 519.0 ± 31.7 cases per year (Table 1). The average number of bed-days was 4178.8 ± 216.4, and the average length of stay was 8.07 ± 0.44 days. The average number of deaths was 14.4 ± 1.7 cases per year, and the average case fatality rate was 2.80 ± 0.49%. Compared to 2019, by 2023 there was an increase of 5.5% in the number of treated patients, a decrease in the number of bed-days by 6.8%, a decrease in the average length of stay by 11.7%, a decrease in the number of deaths by 12.5%, and a decrease in the case fatality rate by 17.3%. Time trend analysis showed a significant downward trend in mean length of stay (r_s_ = −0.90) and mortality (r_s_ = −0.90) over the study period.

Over the period 2019–2023, chest injuries without internal organ involvement were the most common thoracic pathology group, accounting for 50.9% of the 2152 cases included in the pathology-specific analysis (Table 2). Lung and pleural injuries ranked second (27.1%), while other chest injuries and purulent-inflammatory diseases accounted for 13.0% and 8.9%, respectively. During the observation period, there was a 31.3% increase in lung and pleural injuries, while the number of other chest injuries decreased by 75.5%. An analysis of the pathology structure revealed statistically significant changes in the distribution of nosological groups by year (χ^2^ = 158.8; *p* < 0.001). Data for the age groups of children under 1 year and children aged 1–14 years are not available. Values are presented as *n* (%). Percentages in parentheses indicate the proportion of cases occurring in each study year relative to the total number of cases within the corresponding pathology group during 2019–2023.

A total of 262 patients participated in the survey, including 158 (60.3%) males and 104 (39.7%) females. The age distribution was comparable between genders (χ^2^ = 1.528, *p* = 0.910), with the largest proportions of participants aged 30–39 years (22.9%) (Table 3). Overall, patients reported favorable experiences with emergency thoracic surgical care. Although 51.0% indicated that the time to diagnosis exceeded six hours, 57.6% rated the timeliness of diagnostic procedures as very prompt or prompt. The quality of diagnostic procedures was rated as good or excellent by 68.3% of patients, while 96.2% reported that diagnostic results were clearly explained or that their questions were fully answered. Surgical treatment was provided to 67.7% of respondents, 80.5% rated treatment as effective or very effective, and 82.4% reported no treatment -related complications. Furthermore, 84.9% of patients reported receiving regular information about their disease and treatment. Differences between male and female patients were generally not observed across questionnaire items. Statistically significant differences were identified only for treatment type (χ^2^ = 9.659, *p* = 0.008; Cramer’s V = 0.193) and the provision of information regarding disease and treatment (χ^2^ = 9.097, *p* = 0.011; Cramer’s V = 0.187). In both cases, the effect size was small, indicating modest differences between male and female patients.

A total of 123 healthcare professionals participated in the survey, including 104 (84.6%) males and 19 (15.4%) females (Table 4). The distributions of age, years of professional experience, qualification category, frequency of emergency thoracic surgical care, and thoracic surgery specialization were comparable between genders (all *p* > 0.05). Overall, healthcare professionals evaluated the organization of emergency thoracic surgical care positively. Most respondents considered patient transportation to be timely (81.0%), available resources to be fully adequate or generally adequate (87.4%), staff qualifications to be highly or adequately sufficient (75.6%), and interdepartmental coordination to be effective (86.5%). Preoperative diagnosis and postoperative care were also positively assessed by the majority of respondents (88.1% and 84.3%, respectively). Chest trauma was identified as the leading indication for emergency thoracic surgical care (66.1%), followed by acute thoracic conditions such as pneumothorax and empyema (23.7%). The most frequently reported organizational challenges were insufficient equipment (34.1%) and limited time for diagnosis (30.1%). The most commonly proposed improvements included improved department equipment (61.8%), enhanced staff training (58.5%), increased funding (45.5%), and specialized training programs (41.5%). Significant gender-related differences were observed for academic degree distribution (χ^2^ = 20.390, *p* = 0.001; Cramer’s V = 0.67, large effect) and the perceived main reason for emergency thoracic surgical care (χ^2^ = 15.387, *p* = 0.002; Cramer’s V = 0.36, moderate effect). No significant gender differences were identified for the remaining organizational indicators.

Multivariable ordinal logistic regression was performed to examine factors associated with overall satisfaction with the organization of treatment (Table 5 and Table 6). Of the 262 patients included in the survey, 255 had complete data for all variables entered into the model. After adjustment for age, gender, time to diagnosis, treatment type, and treatment-related complications, age and gender were not significantly associated with overall satisfaction. Compared with surgical treatment, conservative treatment was associated with lower odds of reporting a higher satisfaction category (aOR = 0.21; 95% CI: 0.07–0.61; *p* = 0.004), as was medication therapy (aOR = 0.15; 95% CI: 0.05–0.42; *p* < 0.001). Patients who experienced treatment-related complications also had lower odds of reporting a higher satisfaction category (aOR = 0.11; 95% CI: 0.05–0.26; *p* < 0.001). Time to diagnosis was not statistically significant after adjustment (aOR = 0.72; 95% CI: 0.48–1.09; *p* = 0.122). For the healthcare professional survey, multivariable ordinal logistic regression was used to examine factors associated with the overall assessment of the organization of emergency care. Complete data were available for 117 respondents. After adjustment for sex, age, professional experience, qualification category, and thoracic surgery specialization, none of these characteristics showed a statistically significant independent association with the overall organizational assessment. Female sex was not independently associated with the outcome (aOR = 1.21; 95% CI: 0.41–3.53; *p* = 0.732), and thoracic surgery specialization was also not significant (aOR = 1.41; 95% CI: 0.61–3.25; *p* = 0.421). A sensitivity analysis using binary logistic regression was performed after dichotomizing overall satisfaction as fully satisfied versus partially/not satisfied. The results were consistent with the primary ordinal regression model. Conservative treatment (aOR = 0.24; 95% CI: 0.08–0.69; *p* = 0.009), medication therapy (aOR = 0.19, 95% CI: 0.06–0.54; *p* = 0.002), and treatment-related complications (aOR = 0.13, 95% CI: 0.05–0.29; *p* < 0.001) remained significantly associated with lower odds of satisfaction. Age, gender, and time to diagnosis remained non–significant.

**Figure 1:**
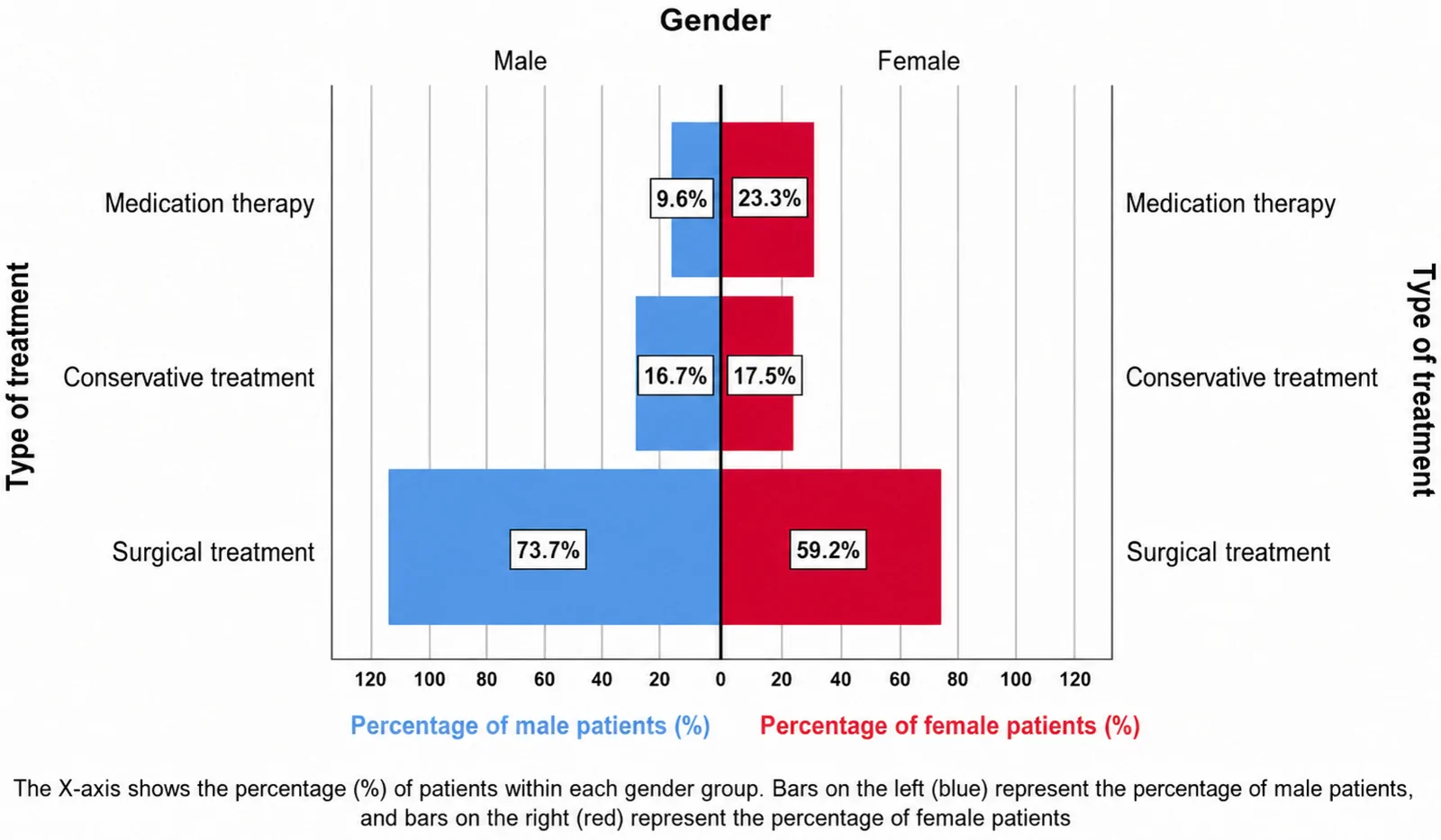
Distribution of treatment types according to gender.

Although statistically significant differences by gender were identified for treatment type and provision of information about disease and treatment, the corresponding effect sizes were small (Cramer’s V = 0.193 and 0.187, respectively), indicating that these differences were modest in magnitude. In the adjusted analysis, treatment type and treatment-related complications showed the strongest associations with overall satisfaction with the organization of treatment. In contrast, age, gender, and time to diagnosis were not independently associated with overall satisfaction after adjustment. As shown in Figure 1, surgical treatment was the predominant management approach in both male and female patients. However, the proportion of patients undergoing surgical treatment was higher among males than females, whereas conservative treatment and medication therapy were relatively more frequent among female patients. These findings are consistent with the statistically significant association between gender and treatment type (χ^2^ = 9.659, *p* = 0.008).

**Figure 2:**
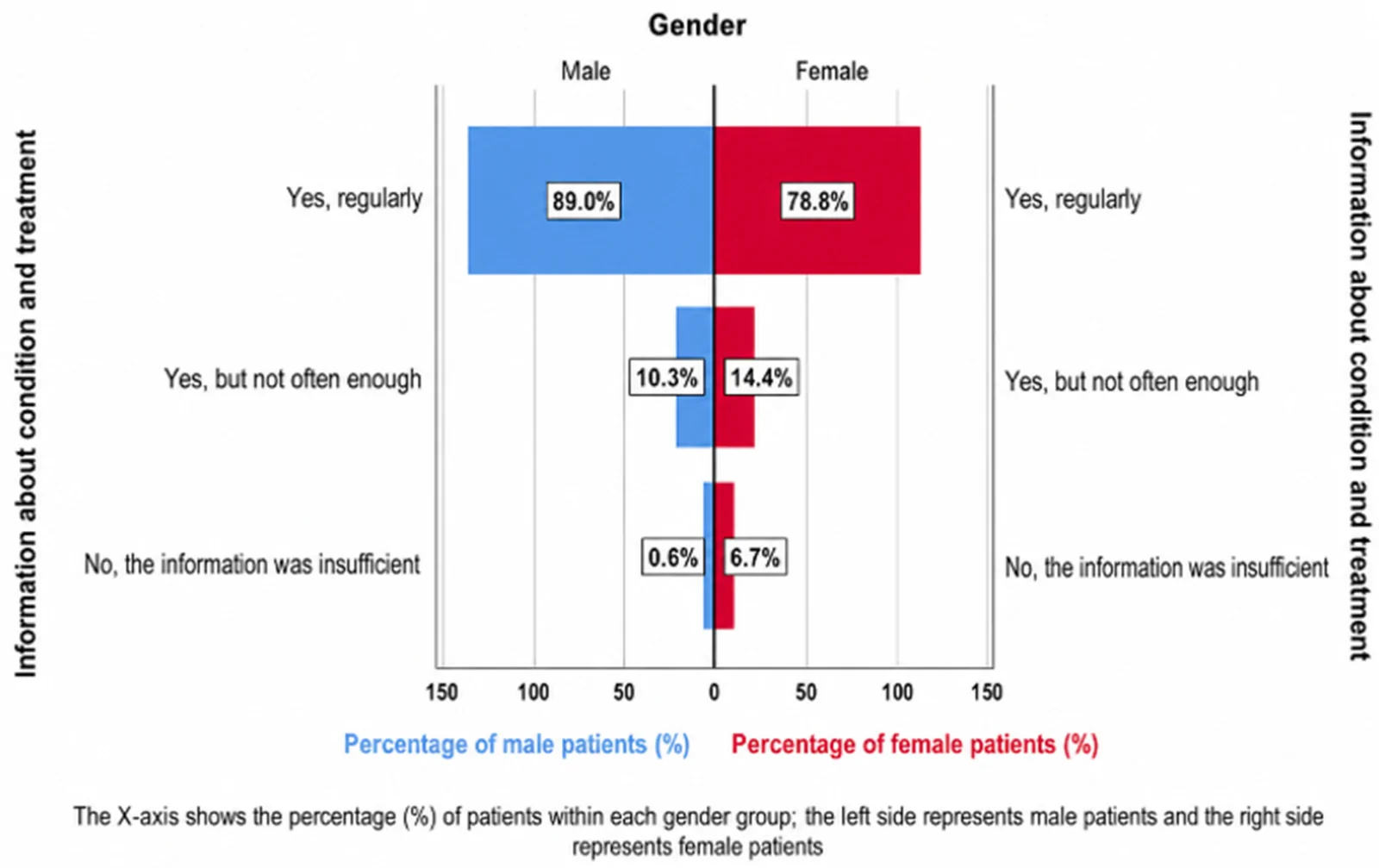
Information provided about the disease and treatment according to gender.

Figure 2 illustrates patients’ assessment of the information provided regarding their disease and treatment. Most respondents in both groups reported receiving regular information from healthcare professionals. Nevertheless, female patients more frequently indicated that the information was insufficient or not provided regularly compared with male patients, corresponding to the statistically significant difference observed between genders (χ^2^ = 9.097, *p* = 0.011).

## 4. Discussion

Emergency thoracic surgical care was examined from both clinical and organizational perspectives by integrating retrospective hospital records with survey data from patients and healthcare professionals. Considering these complementary sources of information allowed the study to characterize not only treatment outcomes but also the effectiveness of healthcare organization, coordination, and service delivery. Rather than assessing clinical outcomes alone, this approach enables the evaluation of organizational performance, patient experience, and barriers affecting service delivery. Overall, the findings suggest that emergency thoracic surgical care in Almaty remained stable throughout the study period, with gradual improvements in hospital outcomes. At the same time, both patient and healthcare professional surveys identified opportunities for further optimization of communication and coordination, and organizational support. Thoracic trauma remained the predominant indication for emergency thoracic surgical care throughout the study period. The findings should be interpreted in terms of both statistical and clinical relevance. Although some differences in patient-reported assessments were statistically significant, the small effect sizes indicate that their practical significance was limited. In the adjusted analysis, treatment type and treatment-related complications showed the strongest associations with overall satisfaction. In contrast, age, gender, and time to diagnosis were not independently associations with this outcome. These associations should be interpreted cautiously, particularly because differences in disease severity and other clinical characteristics could not be fully accounted for. Thoracic trauma remained the predominant indication for emergency thoracic surgical care throughout the study period, which is consistent with previous reports identifying chest trauma as an important component of emergency surgical and trauma care [10,11,12]. Most thoracic injuries can be managed conservatively or with tube thoracostomy; however, a relatively small proportion of patients require emergency thoracic surgery because of life-threatening hemorrhage, major airway injury, cardiac injury, or persistent physiological instability [13]. Consequently, the effectiveness of emergency thoracic services depends not only on surgical expertise but also on rapid diagnosis, multidisciplinary decision-making, and timely access to specialized care. The observed reductions in mortality and length of hospital stay may be consistent with progressive improvements in emergency care pathways; however, the retrospective design does not allow these trends to be attributed to specific organizational changes. In our study, this pattern remained relatively stable over time, suggesting that the observed improvement in hospital outcomes was unlikely to be explained simply by a major change in the overall case structure. The concurrent reduction in mortality and length of hospital stay may instead reflect improvements in care organization and clinical pathways. However, the retrospective design and lack of adjustment for injury severity preclude establishing a causal relationship. Similar improvements in outcomes following structured, protocol-based trauma care have been reported in other settings [14]. The concentration of emergency thoracic surgical activity within major multidisciplinary hospitals in Almaty is also consistent with contemporary approaches to regionalized trauma care. Current recommendations emphasize rapid diagnosis, multidisciplinary decision–making, appropriate patient selection, and access to specialized surgical and intensive care resources [15,16,17]. Our findings provide a local organizational perspective on these recommendations: although overall hospital outcomes improved, healthcare professionals continued to identify diagnostic delays, equipment availability, patient transportation, workforce capacity, and interdepartmental coordination as areas requiring improvement. Thus, favorable trends in hospital outcomes do not necessarily indicate the absence of organizational barriers. Recent evidence underscores the importance of multidisciplinary, well-coordinated systems for thoracic trauma care. The 2025 WSES–AAST guidelines emphasize timely diagnosis, multidisciplinary management, appropriate monitoring, and organized referral pathways for patients requiring specialized care [18]. These recommendations are consistent with our findings, as healthcare professionals identified diagnostic processes, resource availability, patient transportation, and interdepartmental coordination as areas requiring further attention. Recent studies of patient- reported outcomes in emergency surgery have also highlighted communication, treatment experience, and complications as important components of patients’ perception of care [19,20], which is consistent with the associations observed in our patient survey. The patient and healthcare professional surveys also revealed complementary aspects of care organization. While patients generally evaluated the care received positively, healthcare professionals identified persistent limitations in resources, coordination, and service delivery. This difference is important because patient satisfaction and organizational performance reflect different dimensions of healthcare quality. Taken together, the findings suggest that favorable patient experience can coexist with system-level challenges recognized by healthcare professionals, supporting the use of both perspectives when evaluating emergency thoracic surgical services [21,22,23,24,25]. The strengths of the present study include its multicomponent design, integration of hospital and questionnaire data, inclusion of five major multidisciplinary hospitals provide emergency thoracic surgery within the city, and simultaneous evaluation of hospital outcomes, patient experiences, and healthcare professional perspectives. Several limitations should be considered when interpreting the findings. First, the retrospective component was based on routinely collected hospital data and therefore does not permit causal inference about temporal changes. Second, the study was conducted within one metropolitan healthcare system, which may limit the generalizability of the findings to other regions with different organizational structures and resource availability. Selection bias may have affected both questionnaire surveys, as participants who agreed to participate may have differed from those who declined or were unable to respond due to their clinical condition. A similar self-selection effect may have occurred among healthcare professionals who chose to participate. Recall bias was likely limited because patients completed the questionnaire during hospitalization. However, responses regarding the timing of diagnosis, treatment processes, and communication relied on patients’ recollections and personal perception of events during the hospital stay. They may therefore have been subject to some degree of recall error. Response bias should also be considered because the questionnaire data were self-reported. Patients may have been more likely to provide favorable assessments while receiving care in the participating hospitals. In contrast, healthcare professionals may have tended to report socially desirable evaluations of the organization and quality of services in their institutions. Finally, information on injury severity, prehospital management, long-term functional recovery, and health-related quality of life was unavailable, which limited more detailed assessment of factors associated with clinical outcomes. The study was conducted in five hospitals within a single metropolitan healthcare system, which limits the generalizability of the findings to other regions of Kazakhstan with different healthcare infrastructure, resource availability, and organization of emergency surgical services. Future multicenter studies involving hospitals from different regions are needed to determine whether these findings apply to other healthcare settings. Information on injury severity and comorbidities was not systematically available and therefore could not be included in the multivariable analysis. These factors may influence treatment selection, clinical course, complications, and patient-reported satisfaction. Therefore, residual confounding cannot be excluded. In particular, the observed association between treatment type and satisfaction should not be interpreted as evidence of a causal effect of treatment modality. Future studies should incorporate standardized measures of injury severity and comorbidity burden when evaluating predictors of outcomes. Missing responses resulted in small variations in the number of observations across questionnaire items, and the multivariable patient analysis was restricted to 255 of 262 participants with complete data. Although the proportion of excluded patients was small (2.7%), bias due to missing data cannot be completely ruled out if missingness was associated with participant characteristics or questionnaire responses.

## 5. Conclusions

This study provides a multicomponent assessment of emergency thoracic surgical care in five multidisciplinary hospitals in Almaty. During the study period, mortality and length of hospital stay decreased, while patient-reported assessments were generally favorable. Treatment type and treatment-related complications were associated with overall satisfaction, and healthcare professionals identified several organizational areas requiring further attention. Further multicenter studies are needed to confirm the findings and assess their applicability to other healthcare settings.

## Figures and Tables

**Table 1:** Dynamics of indicators of emergency specialized thoracic care in 2019–2023.

Year	Hospitalized Patients	Bed Days	Average Length of Hospital Stays (Days)	Died	Mortality Rate (%)
2019	512	4475	8.74	16	3.13
2020	473	3872	8.19	16	3.38
2021	514	4183	8.14	14	2.72
2022	556	4195	7.54	12	2.16
2023	540	4169	7.72	14	2.59
Spearman’s correlation coefficient (r_s_)	+0.70	−0.30	−0.90	−0.82	−0.90

**Table 2:** Dynamics of the main groups of thoracic pathology.

Pathology Group	ICD-10	2019	2020	2021	2022	2023	Total, *n* (%)	Share, %	Mean ± SD	Change, %
Chest injuries without injury to internal organs	S20-S26	223 (20.3)	200 (18.2)	212 (19.3)	251 (22.9)	211 (19.2)	1097	50.9	219.4 ± 19.6	−5.4
Lung and pleural injuries	S27.0	115 (19.7)	98 (16.8)	85 (14.6)	135 (23.1)	151 (25.9)	584	27.1	116.8 ± 27.6	+31.3
Other chest injuries	S28-S29	143 (51.0)	34 (12.1)	40 (14.3)	28 (10.0)	35 (12.5)	280	13.0	56.0 ± 50.5	−75.5
Purulent-inflammatory diseases	J85-J86	31 (16.2)	35 (18.3)	50 (26.2)	41 (21.5)	34 (17.8)	191	8.9	38.2 ± 7.8	+9.7
Total	-	512	367	387	455	431	2152	100.0	-	-

Differences in the distribution of the main groups of thoracic pathology across the study years were statistically significant (x^2^ = 158.8; df = 12; *p* < 0.001). Table 2 includes the main thoracic pathology groups selected for pathology-specific analysis (*n* = 2152). An additional 443 hospitalizations recorded during the study period were related to other thoracic diagnoses or pathology groups and are not included in this table. Percentages in the “Share” column were calculated using the 2152 cases presented in the table as the denominator.

**Table 3:** Demographic characteristics and patient-reported assessment of emergency thoracic surgical care.

Questions	Answers	Male, *n* (%)	Female, *n* (%)	Total, *n* (%)	χ^2^	*p*-Value
Age	18–29	21 (13.3)	16 (15.4)	37 (14.1)		
	30–39	37 (23.4)	23 (22.1)	60 (22.9)		
	40–49	20 (12.7)	15 (14.4)	35 (13.4)		
	50–59	18 (11.4)	15 (14.4)	33 (12.6)		
	60–69	24 (15.2)	15 (14.4)	39 (14.9)		
	70 years and older	38 (24.1)	20 (19.2)	58 (22.1)		
	Total	158 (100.0)	104 (100.0)	262 (100.0)	1.528	0.910
Time to diagnosis	Less than 1 h	24 (15.5)	21 (20.2)	45 (17.4)		
	1–3 h	22 (14.2)	18 (17.3)	40 (15.4)		
	3–6 h	25 (16.1)	17 (16.3)	42 (16.2)		
	More than 6 h	84 (54.2)	48 (46.2)	132 (51.0)		
	Total	155 (100.0)	104 (100.0)	259 (100.0)	1.976	0.577
Timeliness of diagnostic procedures	Very prompt	27 (17.1)	19 (18.3)	46 (17.6)		
	Prompt	65 (41.1)	40 (38.5)	105 (40.1)		
	Acceptable	53 (33.5)	38 (36.5)	91 (34.7)		
	Delayed	11 (7.0)	6 (5.8)	17 (6.5)		
	Very delayed	2 (1.3)	1 (1.0)	3 (1.1)		
	Total	158 (100.0)	104 (100.0)	262 (100.0)	0.512	0.972
Explanation of the diagnostic results	Everything was clear; no further questions	79 (50.3)	36 (34.6)	115 (44.1)		
	Had questions; fully answered	74 (47.1)	62 (59.6)	136 (52.1)		
	Had questions; answers were unclear	3 (1.9)	4 (3.8)	7 (2.7)		
	Questions remained unanswered	1 (0.6)	2 (1.9)	3 (1.1)		
	Total	157 (100.0)	104 (100.0)	261 (100.0)	7.145	0.067
Quality of diagnostic procedures	Very poor	3 (1.9)	0 (0.0)	3 (1.1)		
	Poor	2 (1.3)	7 (6.7)	9 (3.4)		
	Satisfactory	45 (28.5)	26 (25.0)	71 (27.1)		
	Good	83 (52.5)	54 (51.9)	137 (52.3)		
	Excellent	25 (15.8)	17 (16.3)	42 (16.0)		
	Total	158 (100.0)	104 (100.0)	262 (100.0)	7.584	0.108
Treatment explanation	Fully explained and easy to understand	138 (87.3)	90 (86.5)	228 (87.0)		
	Partially explained	17 (10.8)	10 (9.6)	27 (10.3)		
	Not explained	3 (1.9)	4 (3.8)	7 (2.7)		
	Total	158 (100.0)	104 (100.0)	262 (100.0)	1.172	0.557
Type of treatment	Surgical treatment	115 (73.7)	61 (58.7)	176 (67.7)		
	Conservative treatment	26 (16.7)	18 (17.3)	44 (16.9)		
	Medication therapy	15 (9.6)	25 (24.0)	40 (15.4)		
	Total	156 (100.0)	104 (100.0)	260 (100.0)	9.659	**0.008**
Treatment effectiveness	Very effective	39 (24.8)	29 (27.9)	68 (26.0)		
	Effective	91 (58.0)	51 (49.0)	142 (54.2)		
	Satisfactory	22 (14.0)	18 (17.3)	40 (15.3)		
	Somewhat ineffective	4 (2.5)	5 (4.8)	9 (3.4)		
	Ineffective	1 (0.6)	1 (1.0)	2 (0.8)		
	Total	157 (100.0)	104 (100.0)	261 (100.0)	2.594	0.628
Treatment-related side effects or complications	Yes, serious	6 (3.8)	4 (3.8)	10 (3.8)		
	Yes, minor	20 (12.7)	16 (15.4)	36 (13.7)		
	No	132 (83.5)	84 (80.8)	216 (82.4)		
	Total	158 (100.0)	104 (100.0)	262 (100.0)	0.398	0.819
Frequency of medical rounds	Very often (more than twice a day)	66 (42.2)	32 (30.8)	98 (37.7)		
	Often (1–2 times a day)	70 (44.9)	53 (51.0)	123 (47.3)		
	Once a day	14 (9.0)	12 (11.5)	26 (10.0)		
	Rarely (less than once a day)	5 (3.2)	7 (6.7)	12 (4.6)		
	Not at all	1 (0.6)	0 (0.0)	1 (0.4)		
	Total	156 (100.0)	104 (100.0)	260 (100.0)	5.451	0.244
Pain or discomfort level	No pain	54 (34.1)	29 (27.9)	83 (31.7)		
	Mild pain	64 (40.5)	41 (39.4)	105 (40.1)		
	Moderate pain	37 (23.4)	31 (29.8)	68 (26.0)		
	Severe pain	2 (1.3)	3 (2.9)	5 (1.9)		
	Very severe pain	1 (0.6)	0 (0.0)	1 (0.4)		
	Total	158 (100.0)	104 (100.0)	262 (100.0)	3.308	0.508
Time for improvement	Immediately	8 (5.1)	3 (2.9)	11 (4.2)		
	Within the first day	29 (18.5)	21 (20.2)	50 (19.1)		
	Within 2–3 days	102 (65.0)	67 (64.4)	169 (64.5)		
	After one week or later	18 (11.5)	11 (10.6)	29 (11.1)		
	No improvement	1 (0.6)	1 (1.0)	2 (0.8)		
	Total	157 (100.0)	104 (100.0)	261 (100.0)	0.943	0.918
Quality of patient support services	Very poor	1 (0.6)	0 (0.0)	1 (0.4)		
	Poor	5 (3.2)	6 (5.8)	11 (4.2)		
	Satisfactory	37 (23.9)	28 (26.9)	65 (24.9)		
	Good	93 (60.0)	56 (53.8)	149 (57.1)		
	Excellent	19 (12.3)	14 (13.5)	33 (12.6)		
	Total	155 (100.0)	104 (100.0)	259 (100.0)	2.330	0.675
Information provided about the disease and treatment	Yes, regularly	138 (89.0)	82 (78.8)	220 (84.9)		
	Yes, but not often enough	16 (10.3)	15 (14.4)	31 (12.0)		
	No, the information was insufficient	1 (0.6)	7 (6.7)	8 (3.1)		
	Total	155 (100.0)	104 (100.0)	259 (100.0)	9.097	**0.011**

Data are presented as *n* (%). Percentages were calculated based on the number of respondents who answered each question. Missing responses were excluded from the analysis of the corresponding item. Pearson’s χ^2^ test was used to compare categorical variables. A two-sided *p*-value < 0.05 was considered statistically significant. Statistically significant *p*-values are shown in bold.

**Table 4:** Assessment of the organization of emergency thoracic surgical care in a metropolitan multidisciplinary hospital by medical staff.

Questions	Answers	Male, *n* (%)	Female, *n* (%)	Total, *n* (%)	Statistical Test	*p*-Value
Age	Under 30 years	13 (12.5)	5 (26.3)	18 (14.6)		
	30–40 years	43 (41.3)	6 (31.6)	49 (39.8)		
	40–50 years	29 (27.9)	3 (15.8)	32 (26.0)		
	Over 50 years	19 (18.3)	5 (26.3)	24 (19.5)		
	Total	104 (100.0)	19 (100.0)	123 (100.0)	3.917	0.271
Years of experience in medicine	Less than 5 years	16 (15.4)	6 (31.6)	22 (17.9)		
	5–10 years	23 (22.1)	3 (15.8)	26 (21.1)		
	10–20 years	31 (29.8)	5 (26.3)	36 (29.3)		
	More than 20 years	34 (32.7)	5 (26.3)	39 (31.7)		
	Total	104 (100.0)	19 (100.0)	123 (100.0)	2.933	0.402
Professional qualification category	Second qualification category	12 (11.5)	2 (10.5)	14 (11.4)		
	First qualification category	13 (12.5)	2 (10.5)	15 (12.2)		
	Highest qualification category	45 (43.3)	6 (31.6)	51 (41.5)		
	No qualification category	34 (32.7)	9 (47.4)	43 (35.0)		
	Total	104 (100.0)	19 (100.0)	123 (100.0)	1.585	0.663
Academic degree	Candidate of Medical Sciences	6 (14.3)	0 (0.0)	6 (13.0)		
	Doctor of Medical Sciences	2 (4.8)	3 (75.0)	5 (10.9)		
	Master of Medical Sciences	29 (69.0)	0 (0.0)	29 (63.0)		
	PhD	5 (11.9)	1 (25.0)	6 (13.0)		
	Total	42 (100.0)	4 (100.0)	46 (100.0)	20.390	**0.001**
Frequency of emergency thoracic surgical care	Less than once a month	31 (31.0)	4 (23.5)	35 (29.9)		
	1–2 times a month	22 (22.0)	3 (17.6)	25 (21.4)		
	Once a week	21 (21.0)	2 (11.8)	23 (19.7)		
	Several times a week	26 (26.0)	8 (47.1)	34 (29.1)		
	Total	100 (100.0)	17 (100.0)	117 (100.0)	3.248	0.355
Main reason for emergency thoracic surgical care	Chest trauma (road traffic accidents, falls)	69 (69.0)	9 (50.0)	78 (66.1)		
	Acute thoracic conditions (pneumothorax, empyema)	25 (25.0)	3 (16.7)	28 (23.7)		
	Oncological complications	1 (1.0)	3 (16.7)	4 (3.4)		
	Other	5 (5.0)	3 (16.7)	8 (6.8)		
	Total	100 (100.0)	18 (100.0)	118 (100.0)	15.387	**0.002**
Timeliness of patient transport	Always timely	33 (32.0)	7 (38.9)	40 (33.1)		
	Timely in most cases	48 (46.6)	10 (55.6)	58 (47.9)		
	Sometimes delayed	21 (20.4)	1 (5.6)	22 (18.2)		
	Often delayed	1 (1.0)	0 (0.0)	1 (0.8)		
	Total	103 (100.0)	18 (100.0)	121 (100.0)	2.503	0.475
Availability of resources for emergency thoracic care	Fully adequate	40 (39.6)	8 (44.4)	48 (40.3)		
	Adequate, but occasional shortages	47 (46.5)	9 (50.0)	56 (47.1)		
	Insufficient equipment and medications	14 (13.9)	1 (5.6)	15 (12.6)		
	Critical shortage	0 (0.0)	0 (0.0)	0 (0.0)		
	Total	101 (100.0)	18 (100.0)	119 (100.0)	0.964	0.618
Staff qualification for emergency thoracic care	Highly qualified	29 (28.7)	6 (33.3)	35 (29.4)		
	Adequately qualified	45 (44.6)	10 (55.6)	55 (46.2)		
	Additional training needed	26 (25.7)	2 (11.1)	28 (23.5)		
	Insufficiently qualified	1 (1.0)	0 (0.0)	1 (0.8)		
	Total	101 (100.0)	18 (100.0)	119 (100.0)	2.079	0.556
Interdepartmental coordination	Very effective	30 (29.7)	5 (27.8)	35 (29.4)		
	Fairly effective	58 (57.4)	10 (55.6)	68 (57.1)		
	Needs improvement	13 (12.9)	3 (16.7)	16 (13.4)		
	Ineffective	0 (0.0)	0 (0.0)	0 (0.0)		
	Total	101 (100.0)	18 (100.0)	119 (100.0)	0.192	0.908
Quality of preoperative diagnosis	Fully satisfactory	36 (36.0)	9 (50.0)	45 (38.1)		
	Satisfactory in most cases	51 (51.0)	8 (44.4)	59 (50.0)		
	Frequent difficulties	13 (13.0)	1 (5.6)	14 (11.9)		
	Unsatisfactory	0 (0.0)	0 (0.0)	0 (0.0)		
	Total	100 (100.0)	18 (100.0)	118 (100.0)	1.628	0.443
Quality of postoperative care	Fully satisfactory	30 (30.9)	9 (50.0)	39 (33.9)		
	Satisfactory in most cases	52 (53.6)	6 (33.3)	58 (50.4)		
	Improvements in care and monitoring are needed	15 (15.5)	3 (16.7)	18 (15.7)		
	Unsatisfactory	0 (0.0)	0 (0.0)	0 (0.0)		
	Total	97 (100.0)	18 (100.0)	115 (100.0)	2.880	0.237
Common challenges in organizing emergency thoracic surgical care	Limited time for diagnosis	30 (28.8)	7 (36.8)	37 (30.1)	0.182	0.670
	Insufficient equipment	38 (36.5)	4 (21.1)	42 (34.1)	Fisher’s exact test	0.293
	Delayed patient transportation	26 (25.0)	8 (42.1)	34 (27.6)	1.573	0.210
	Shortage of qualified staff	26 (25.0)	3 (15.8)	29 (23.6)	Fisher’s exact test	0.559
	Other	0 (0.0)	0 (0.0)	0 (0.0)	Fisher’s exact test	1.000
Thoracic surgery specialization	Yes	36 (36.0)	4 (22.2)	40 (33.9)		
	No	64 (64.0)	14 (77.8)	78 (66.1)		
	Total	100 (100.0)	18 (100.0)	118 (100.0)	1.292	0.256
Suggested improvements in emergency thoracic surgical care	Improved department equipment	65 (62.5)	11 (57.9)	76 (61.8)	0.144	0.704
	Enhanced training of medical staff	61 (58.7)	11 (57.9)	72 (58.5)	0.004	0.951
	Improved interdepartmental coordination	38 (36.5)	5 (26.3)	43 (35.0)	0.738	0.390
	Increased funding	46 (44.2)	10 (52.6)	56 (45.5)	0.457	0.499
	Specialized training programs for staff	41 (39.4)	10 (52.6)	51 (41.5)	1.155	0.283
Overall organization of emergency care	Very effective	22 (22.0)	5 (27.8)	27 (22.9)		
	Effective, but with some shortcomings	65 (65.0)	10 (55.6)	75 (63.6)		
	Needs significant improvement	13 (13.0)	3 (16.7)	16 (13.6)		
	Ineffective	0 (0.0)	0 (0.0)	0 (0.0)		
	Total	100 (100.0)	18 (100.0)	118 (100.0)	0.588	0.745

Data are presented as *n* (%). Percentages were calculated based on the number of respondents who answered each question. Missing responses were excluded from the analysis of the corresponding item. For multiple-response questions (Questions 13 and 15), percentages were calculated using the total number of respondents in each gender group as the denominator; therefore, percentages may exceed 100%. Pearson’s χ^2^ test was used for categorical comparisons; Fisher’s exact test was applied when expected cell counts were <5. A two-sided *p* value < 0.05 was considered statistically significant. Statistically significant *p* values are shown in bold.

**Table 5:** Multivariable ordinal logistic regression analysis of questionnaire outcomes. Patient survey: factors associated with overall satisfaction with the organization of treatment (*n* = 255).

Variable	Adjusted OR	95% CI	*p*-Value
Age category (per category increase)	1.22	0.92–1.62	0.166
Female gender (ref: male)	0.60	0.26–1.39	0.233
Time to diagnosis (per category increase)	0.72	0.48–1.09	0.122
Conservative treatment (ref: surgical treatment)	0.21	0.07–0.61	0.004
Medication therapy (ref: surgical treatment)	0.15	0.05–0.42	<0.001
Treatment-related complications (ref: absent)	0.11	0.05–0.26	<0.001

The dependent variable was overall satisfaction with the organization of treatment, with higher categories representing greater satisfaction. The model was simultaneously adjusted for age, sex, time to diagnosis, treatment type, and treatment-related complications. Age category and time to diagnosis were entered into the model as ordinal variables; therefore, the corresponding adjusted ORs represent the change in the odds of reporting a higher satisfaction category for each one-category increase in age or time to diagnosis, respectively. Surgical treatment was used as the reference category for treatment type. Treatment-related complications were categorized as present (minor or serious) versus absent. The analysis was performed using complete cases; 255 of the 262 patients had complete data for all variables included in the model. Adjusted ORs greater than 1 indicate higher odds of reporting a more favorable satisfaction category, whereas ORs below 1 indicate lower odds.

**Table 6:** Multivariable ordinal logistic regression analysis of questionnaire outcomes. Healthcare professional survey: factors associated with overall assessment of the organization of emergency care (*n* = 117).

Variable	Adjusted OR	95% CI	*p*-Value
Age category (per category increase)	0.85	0.40–1.81	0.679
Female gender (ref: male)	1.21	0.41–3.53	0.732
Professional experience (per category increase)	1.28	0.56–2.91	0.555
Qualification category (per category increase)	0.85	0.50–1.45	0.560
Thoracic surgery specialization (ref: No)	1.41	0.61–3.25	0.421

Multivariable ordinal logistic regression was used for both analyses. For the patient survey, the dependent variable was overall satisfaction with the organization of treatment, with higher categories indicating greater satisfaction. The model was adjusted simultaneously for age, sex, time to diagnosis, type of treatment, and treatment-related complications. Surgical treatment was used as the reference category for treatment type. For the healthcare professional survey, the dependent variable was the overall assessment of the organization of emergency care, with higher categories representing a more favorable assessment. The model included age, sex, professional experience, qualification category, and thoracic surgery specialization. Adjusted ORs greater than 1 indicate higher odds of being in a more favorable outcome category, whereas ORs below 1 indicate lower odds.

## Data Availability

All data generated or analyzed during this study are included in this article. Further inquiries can be directed to the corresponding authors.

## Abbreviations

The following abbreviations are used in this manuscript:

ERT	Emergency Resuscitative Thoracotomy
M	Mean values
SD	Standard deviations

## Appendix A

**Table A1:** Age distribution of surgically treated patients in 2019–2023.

Age	2019, *n* (%)	2020, *n* (%)	2021, *n* (%)	2022, *n* (%)	2023, *n* (%)	Total, *n*	Share, %	Mean ± SD	Change, %
15–17 years	6 (46.2)	3 (23.1)	1 (7.7)	3 (23.1)	0 (0.0)	13	0.7	2.6 ± 2.3	−100.0
18–59 years	266 (19.4)	248 (18.1)	264 (19.3)	320 (23.3)	273 (19.9)	1371	72.9	274.2 ± 27.3	+2.6
60–69 years	49 (17.3)	53 (18.7)	52 (18.3)	67 (23.6)	63 (22.2)	284	15.1	56.8 ± 7.8	+28.6
70 years and older	48 (22.5)	29 (13.6)	33 (15.5)	43 (20.2)	60 (28.2)	213	11.3	42.6 ± 11.9	+25.0
Total	369	333	350	433	396	1881	100.0	-	-

Values for individual age groups are presented as *n* (%). Percentages in parentheses represent the proportion of cases in each study year relative to the total number of cases within the corresponding age group over the entire study period (2019–2023). The “Share” column represents the proportion of each age group among all surgically treated patients (*n* = 1881). Differences in the distribution of age groups across study years were not statistically significant (χ^2^ = 20.91; df = 12; *p* = 0.052). Table A1 presents the age distribution of the subset of surgically treated patients (*n* = 1881) and should be distinguished from the total number of hospitalizations reported in Table 1 (*n* = 2595).
